# Delivery of mRNA to platelets using lipid nanoparticles

**DOI:** 10.1038/s41598-018-36910-2

**Published:** 2019-01-24

**Authors:** S. Novakowski, K. Jiang, G. Prakash, C. Kastrup

**Affiliations:** 1 Michael Smith Laboratories, University of British Columbia, Vancouver, BC Canada; 2 Department of Biochemistry and Molecular Biology, University of British Columbia, Vancouver, BC Canada

## Abstract

Platelets are natural delivery vehicles within the blood, carrying and releasing their contents at sites of vasculature damage. Investigating the biology of platelets, and modifying them for new therapeutic uses, is limited by a lack of methods for efficiently transfecting these cells. The ability of four different classes of lipid nanoparticles (LNPs) to deliver mRNA to platelets was compared using confocal microscopy, flow cytometry and quantitative PCR. The amount of mRNA delivered, mechanism of uptake, and extent of platelet activation depended on the LNP formulation and platelet storage conditions. Cationic LNPs (cLNPs) delivered mRNA to the largest percentage of platelets but induced platelet activation. Ionizable cationic LNPs (icLNPs) delivered mRNA to fewer platelets and did not induce activation. Furthermore, mRNA delivered using icLNPs and cLNPs was stable in resting platelets and was released in platelet microparticles under specific conditions. The results demonstrate that mRNA can be delivered to platelets using cLNPs and icLNPs without impairing platelet aggregation or spreading. Optimizing the LNP formulations used here may lead to a transfection agent for platelets that allows for de novo synthesis of exogenous proteins in the future.

## Introduction

Platelets are key players in many physiological and pathological processes, including hemostasis, thrombosis, inflammation and cancer progression^1^. They are able to regulate these diverse processes in part due to their ability to target and selectively release small molecules, nucleic acids, and proteins at sites of vasculature damage^2–4^. Platelet transfusions are used extensively in the clinic to prevent hemorrhage associated with thrombocytopenia, which can be induced by coagulation disorders, trauma, autoimmune disorders and leukemias^5^. The routine use and natural ability of platelets to target sites of vasculature damage suggests modified platelets may be beneficial. Increasing the efficacy, safety, and functionality of platelets would enable them to better treat hemorrhage, and potentially extend the use of platelet transfusions to new directions in cell therapy. However, there are challenges in modifying platelets. Currently, hematopoietic stem cells need to be transfected to increase protein expression in platelets, and the use of such cells in patients requires intensive preclinical procedures or major advances in *ex vivo* platelet culturing and production^6,7^. An alternative approach would be to directly alter donor-derived platelets *ex vivo* prior to transfusion. However, efficient methods for transfecting platelets with mRNA do not exist.

Since platelets are anucleate, modifying protein expression within the mature platelet requires delivery of RNA-based agents. Before the RNA can alter protein levels, it must reach the cytoplasm of the platelet without triggering an innate immune response^8^. It is also important to avoid activation of platelets, which may increase the risk of thrombosis following transfusion of the modified platelets^9^. Platelet activation, including granule release, shape change and aggregation, can be induced by multiple signalling pathways, all of which could be activated by the delivery agent or foreign mRNA^9,10^. We hypothesized that a lipid-based transfection agent could be formulated to efficiently deliver mRNA to platelets without activating them, which would be a first step towards direct transfection of transfusable platelets.

Identifying a suitable class of transfection agents is a necessary preliminary step. A variety of agents exist for RNA delivery, consisting of lipids or synthetic or natural polymers complexed to the RNA^11–13^. While a commercial lipid-based reagent has been used to deliver siRNA and microRNA to platelets^3,14,15^, it is unknown whether this approach can be used to deliver mRNA. We previously showed that platelets can endocytose phosphatidylcholine liposomes containing components of an *in vitro* transcription reaction, however, the synthesized mRNA did not include all the modifications required for translation in mammalian cells^16^. These liposomes also did not include cationic lipids or polymers that are typically used in lipid carriers of RNA to improve their stability, cellular uptake and endosomal escape^11,13,17^. Furthermore, while cationic lipids have high efficiency *in vitro*, a separate class of lipids termed ionizable cationic lipids have improved pharmacokinetics and are used for *in vivo* delivery^11,18^.

Here we tested which classes of transfection agents can deliver mRNA to platelets. We found that nanoparticles containing cationic lipids or ionizable cationic lipids can deliver mRNA to platelets with minimal platelet activation. The mRNA remained stable in resting platelets for several hours following transfection, and a portion of the mRNA was released in platelet microparticles (MPs) depending on the storage conditions and class of lipids used. This provides a first step towards direct transfection of transfusable platelets and highlights the need to develop transfection agents that are specific for platelets’ unique structure and biochemistry.

## Results

### Specific LNP formulations can be internalized by platelets

**Figure 1:**
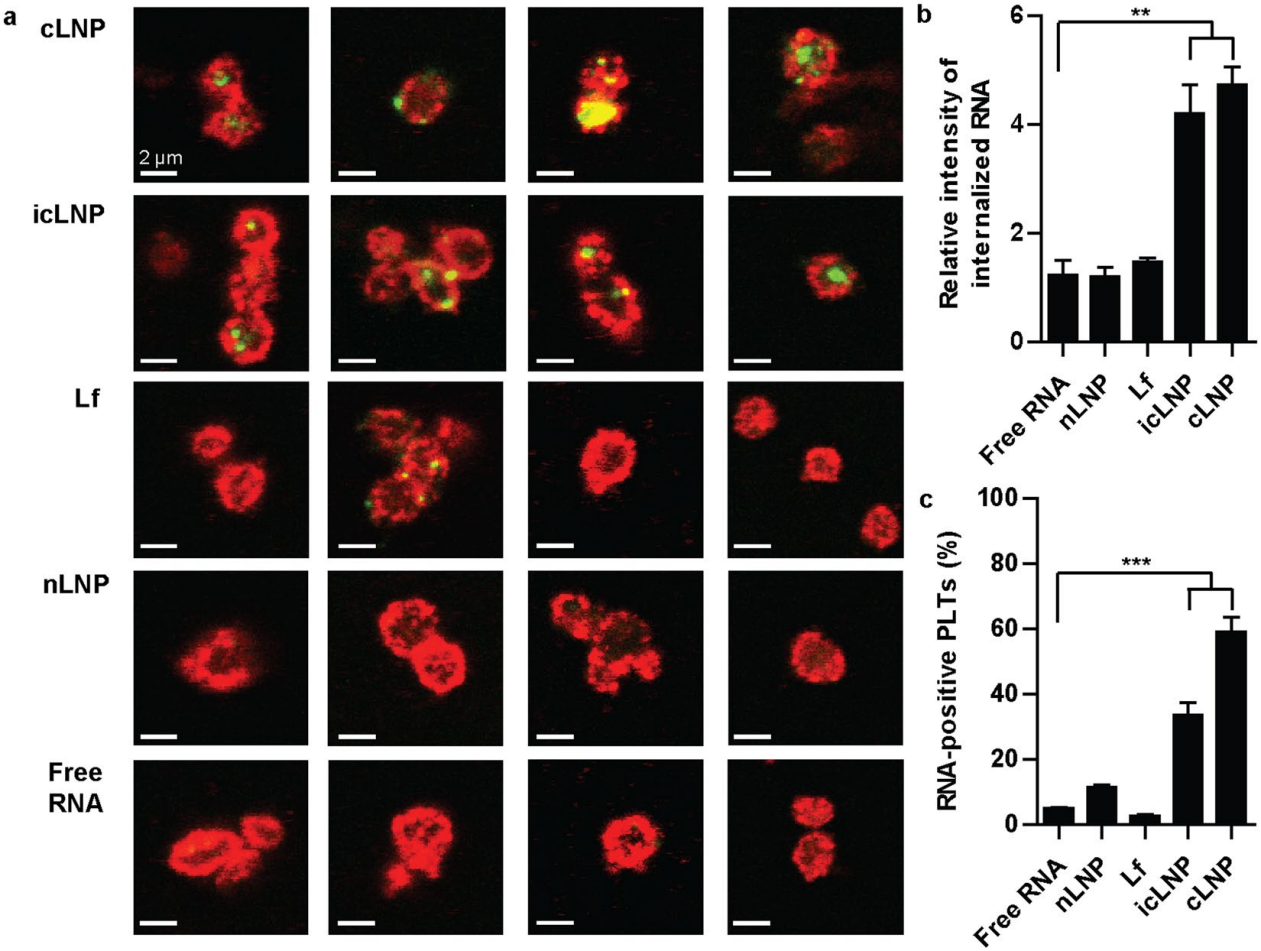
mRNA delivered by icLNPs and cLNPs is internalized. (**a**) Confocal immunofluorescence microscopy of platelets (red) transfected with LNPs containing biotin-labelled RNA (green). Representative images from 4 separate donors are shown. All scale bars indicate 2 µm. (**b**) Quantifying biotin-labelled RNA inside the platelets from images in panel A (n = 4). (**c**) The amount of Cy5-positive platelets transfected with Cy5-mRNA was quantified by flow cytometry (n = 5). Error bars represent SEMs. **P < 0.01, ***P < 0.001.

The amount of mRNA delivered to platelets by four different classes of lipid nanoparticles (LNPs) was quantified using confocal microscopy. The classes were: lipofectamine (Lf), LNPs lacking cationic lipids (nLNPs), LNPs containing a cationic lipid that remains positively charged at physiological pH (cLNPs) and LNPs containing an ionizable cationic lipid that is neutral at physiological pH but becomes positively charged in acidic conditions (icLNPs). All LNPs had an average diameter of 160 to 200 nm, except Lf, which had an average diameter of 350 nm (Supplementary Fig. 1). Lf-mRNA complexes were formed according to the manufacturer’s instructions, to remain comparable with previous studies that transfected platelets with Lf-siRNA^4,14^. While the LNPs were not formed by the same method, an equivalent concentration of RNA for each class of LNP was added to platelet samples during experiments. Preparing platelets with either cLNPs or icLNPs led to an increase in the maximum fluorescence intensity within the cells (P < 0.0001 for cLNPs, P = 0.0003 for icLNPs), but preparing the platelets with free RNA, nLNPs or Lf did not (Fig. 1a,b).

**Figure 2:**
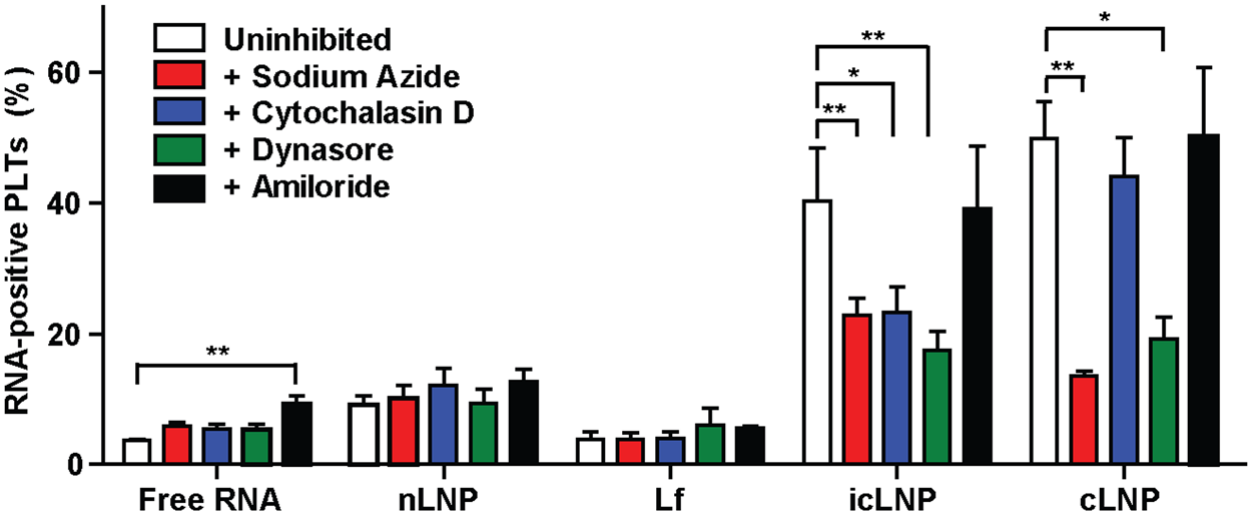
Mechanism of uptake depends on LNP formulation. LNPs encapsulating Cy5-mRNA were delivered to platelets prepared with various endocytosis inhibitors and the percentage of Cy5-positive platelets was quantified by flow cytometry (n = 4–5). Error bars represent SEMs. *P < 0.05, **P < 0.01.

To better understand the mechanism by which LNPs were internalized, Cy5-labelled mRNA was encapsulated in LNPs and the number of Cy5-positive platelets was quantified using flow cytometry. Preparing platelets with either cLNPs or icLNPs led to an increase in the number of Cy5-positive platelets (P < 0.0001, P < 0.0001), while platelets prepared with nLNPs and Lf did not (Fig. 1c). Pre-treating platelets with the metabolic inhibitor sodium azide or the endocytosis inhibitor dynasore^19^ led to a decrease in the percentage of Cy5-positive platelets when prepared with icLNPs (P = 0.0305 for sodium azide, P = 0.0033 for dynasore) or cLNPs (P = 0.0045 for sodium azide, P = 0.0189 for dynasore), but not with free RNA, Lf or nLNPs (Fig. 2). This indicates that dynamin contributes to the uptake of icLNPs and cLNPs. Inhibiting actin polymerization with cytochalasin D^19^ reduced the number of Cy5-positive platelets prepared with icLNPs (P = 0.0383) but not cLNPs, suggesting there are differences in the mechanism of uptake between icLNPs and cLNPs. Inhibiting phagocytosis with amiloride^19^ had no effect on the percentage of Cy5-positive platelets prepared with icLNPs or cLNPs.

### The internalization of LNPs with plaletets depends on initial storage conditons

**Figure 3:**
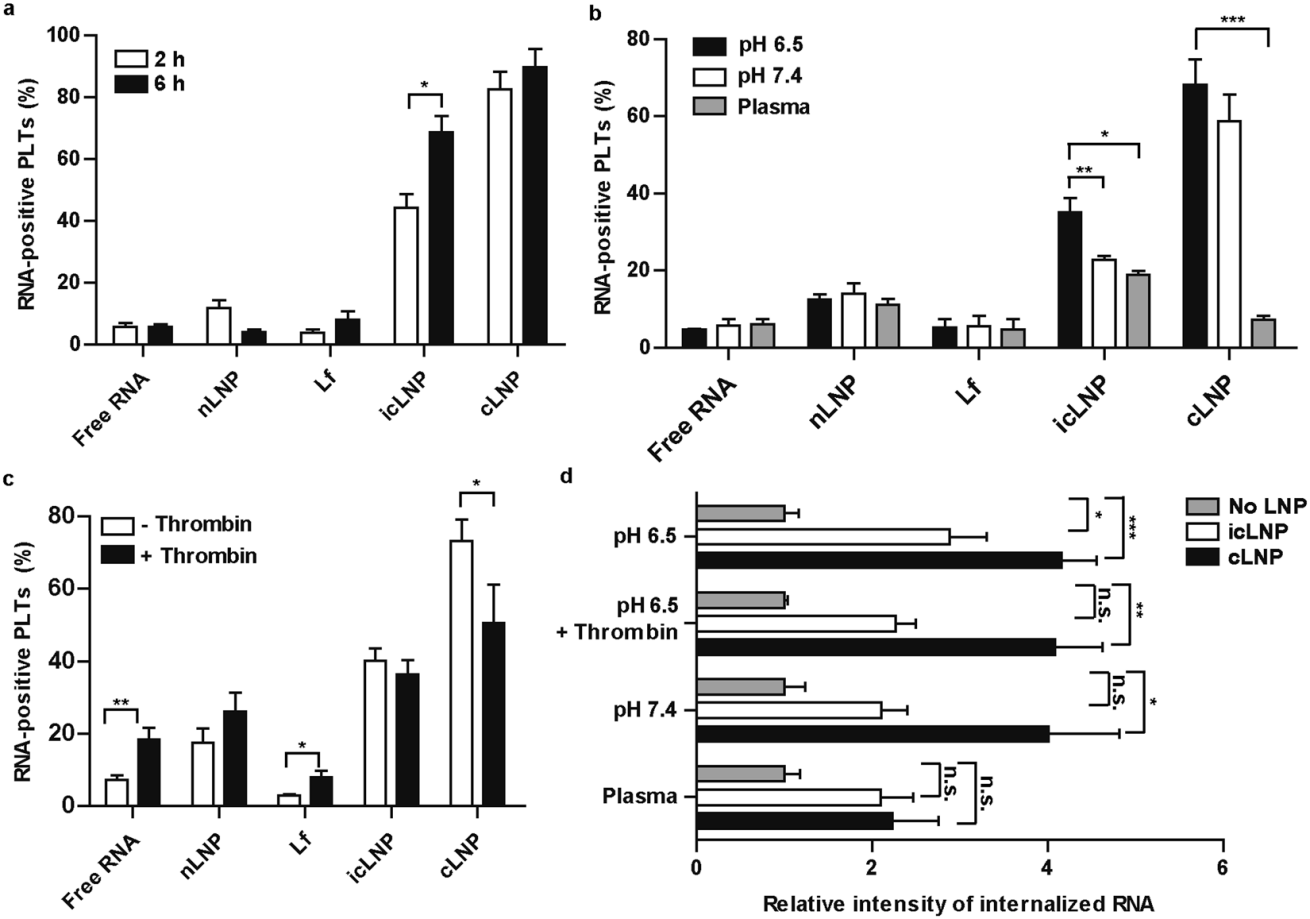
Only the binding of icLNPs to platelets is enhanced with longer incubation while the internalization of both icLNPs and cLNPs is inhibited in plasma. (**a**) LNPs encapsulating Cy5-mRNA were delivered to platelets and Cy5-positive platelets were quantified by flow cytometry (n = 4). (**b**) Comparing binding in Tyrode’s buffer at pH 6.5 or pH 7.4 to plasma (n = 6). (**c**) Binding in the presence and absence of thrombin (n = 10). (**d**) Quantifying biotin-labelled RNA in confocal immunofluorescence images of platelets prepared with LNPs under different storage conditions (n = 5). Error bars represent SEMs. *P < 0.05, **P < 0.01, ***P < 0.001.

Platelet function depends on their storage conditions and whether they have been activated, so the effects of storage time, pH, presence of plasma and presence of a platelet activator during uptake was tested. Increasing the storage time from 2 h to 6 h increased the percentage of Cy5-positive platelets only with icLNPs (P = 0.0158) (Fig. 3a). The percentage of Cy5-positive platelets was also greater in Tyrode’s buffer at pH 6.5 compared to when platelets were stored in plasma for both icLNPs (P = 0.0013) and cLNPs (P < 0.0001) (Fig. 3b). Raising the pH of the Tyrode’s buffer from 6.5 to 7.4 during storage decreased the percentage of Cy5-positive platelets prepared with icLNPs (P = 0.0279) but not cLNPs (Fig. 3b). Activating platelets with thrombin, a potent platelet agonist, prior to adding LNPs, decreased the percentage of Cy5-positive platelets with cLNPs (P = 0.0342) and increased the percentage of Cy5-positive platelets with free RNA (P = 0.0075) or Lf (P = 0.0020), while the percentage of Cy5-positive platelets with nLNPs or icLNPs was unchanged (Fig. 3c).

**Figure 4:**
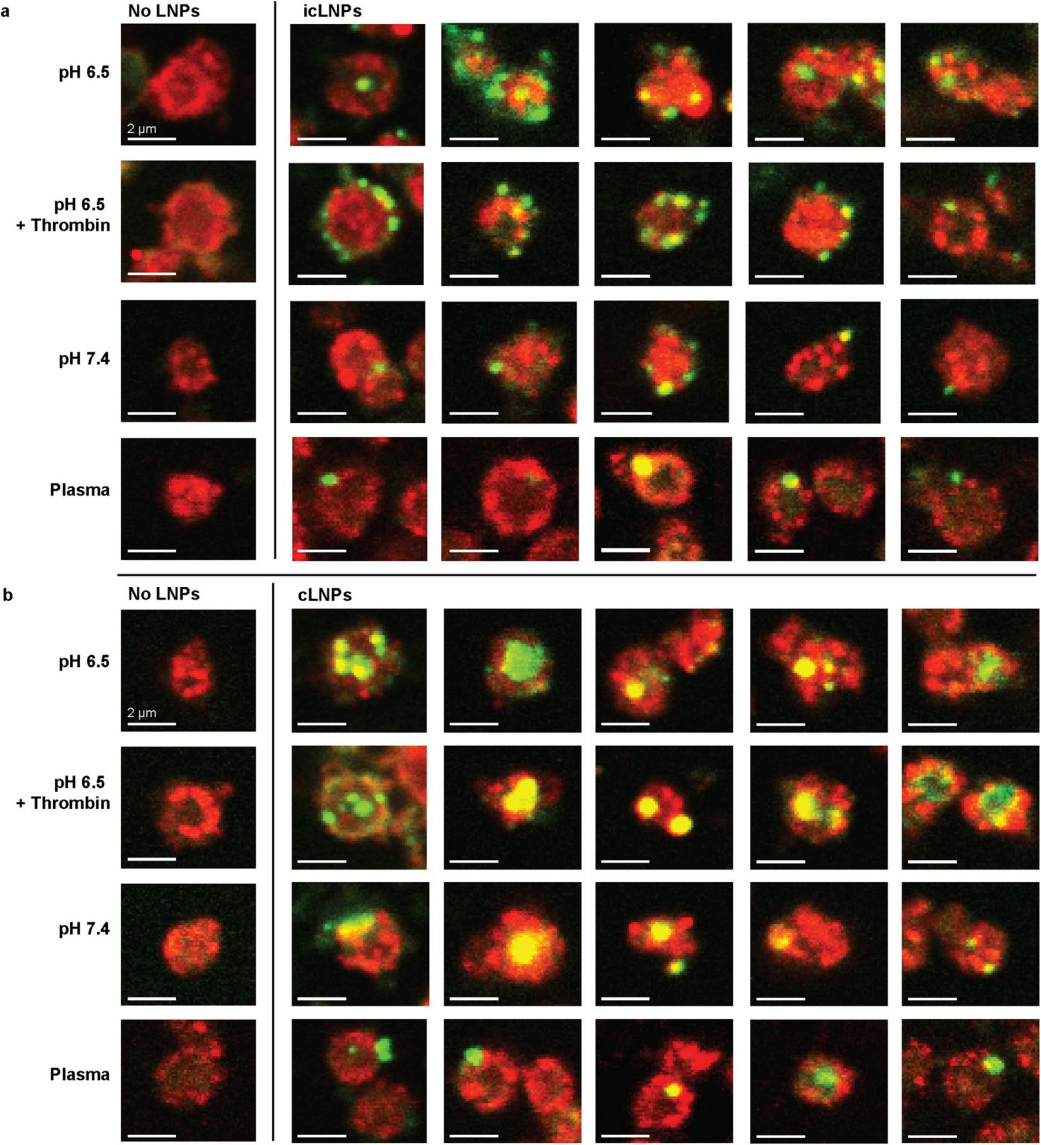
Internalization of icLNPs but not cLNPs depends on initial pH and presence of thrombin. Confocal immunofluorescence microscopy of platelets (red) transfected with (**a**) icLNPs and (**b**) cLNPs containing biotin-labelled RNA (green). Representative images for 5 different donors are shown, with untreated platelets from 2 representative donors for comparison. All scale bars indicate 2 µm.

Confocal microscopy was then used to determine whether internalization of the LNPs occurred under these different storage conditions. Platelets prepared with icLNPs had an increased flourescence intensity inside of the platelets compared to untreated platelets only when unactivated at pH 6.5 (P = 0.0348), and not when activated with thrombin or prepared at pH 7.4 or in plasma (Figs 3d, 4). Platelets prepared with cLNPs had an increased flourescence intensity when prepared at pH 6.5 in the absence (P = 0.0002) or presence of thrombin (P = 0.0108), and at pH 7.4 (p = 0.0198), but not when prepared in plasma (Figs 3d, 4).

### LNPs do not impair platelet function

**Figure 5:**
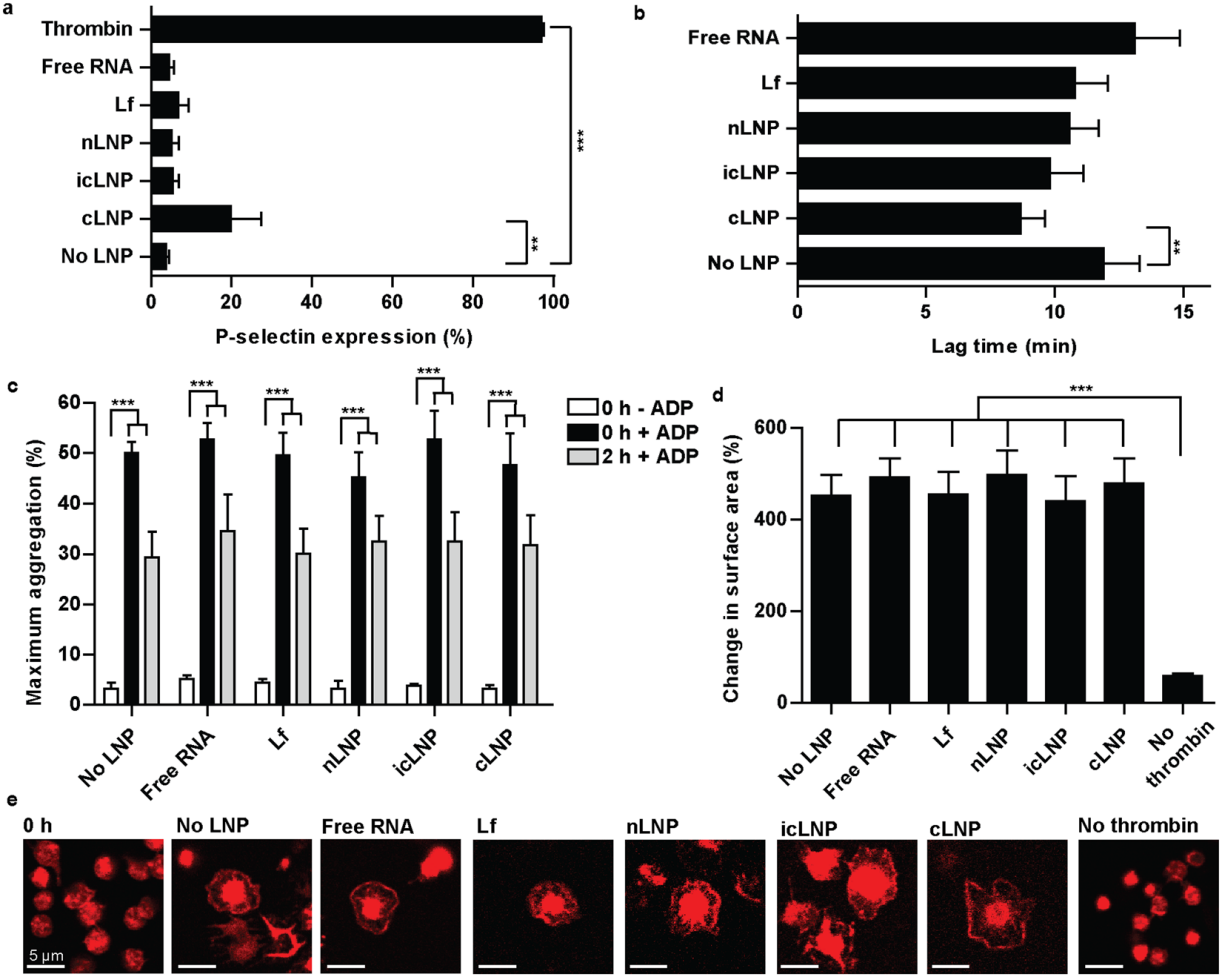
cLNPs activate platelets and the coagulation cascade but do not impair platelet function. (**a**) P-selection expression on platelets, quantified using flow cytometry (n = 4). (**b**) The time for platelets to generate thrombin following treatment with LNPs was quantified using a fluorescent thrombin substrate (n = 10). (**c**) Platelet aggregation was quantified in buffer before and after stimulation with ADP (10 µM), either with no incubation or a 2 h pre-incubation with LNPs (n = 5). (**d**) Quantifying confocal microscopy images of platelets spreading on collagen-coated coverslips following activation with thrombin (n = 5). (**e**) Representative images of spread platelets (n = 5). All scale bars indicate 5 µm. Error bars represent SEMs. *P < 0.05, **P < 0 .01, ***P < 0.001.

To determine whether LNPs activate platelets or inhibit activation, multiple markers of platelet activation and function were assessed. To measure granule release, flow cytometry was used to quantify the expression of P-selectin, which is a membrane-bound protein that becomes exposed to the extracellular environment during activation. Compared to untreated platelets, increased expression of P-selectin occurred only with cLNPs (P = 0.0037) or in controls (P < 0.0001) treated with thrombin (Fig. 5a). To determine if LNPs activated the coagulation cascade, the time to initiate thrombin generation was measured. Thrombin generation was faster in plasma containing platelets prepared with cLNPs compared to untreated platelets (P = 0.0013) (Fig. 5b), consistent with increased activation of platelets. In the absence of platelets, cLNPs alone also decreased the time until thrombin was generated (P = 0.0008) (Supplementary Fig. 2), suggesting that cLNPs can interact directly with components of the coagulation cascade. In contrast, none of the LNPs induced platelet aggregation in buffer, nor did they inhibit platelets from aggregating in response to adenosine diphosphate (ADP) (Fig. 5c). Similarly, LNPs did not inhibit platelets from spreading on a collagen-coated surface in response to thrombin (Fig. 5d,e), suggesting that platelets can still become fully activated after they are prepared with LNPs.

### mRNA is stable in resting platelets and can be released upon activation

**Figure 6:**
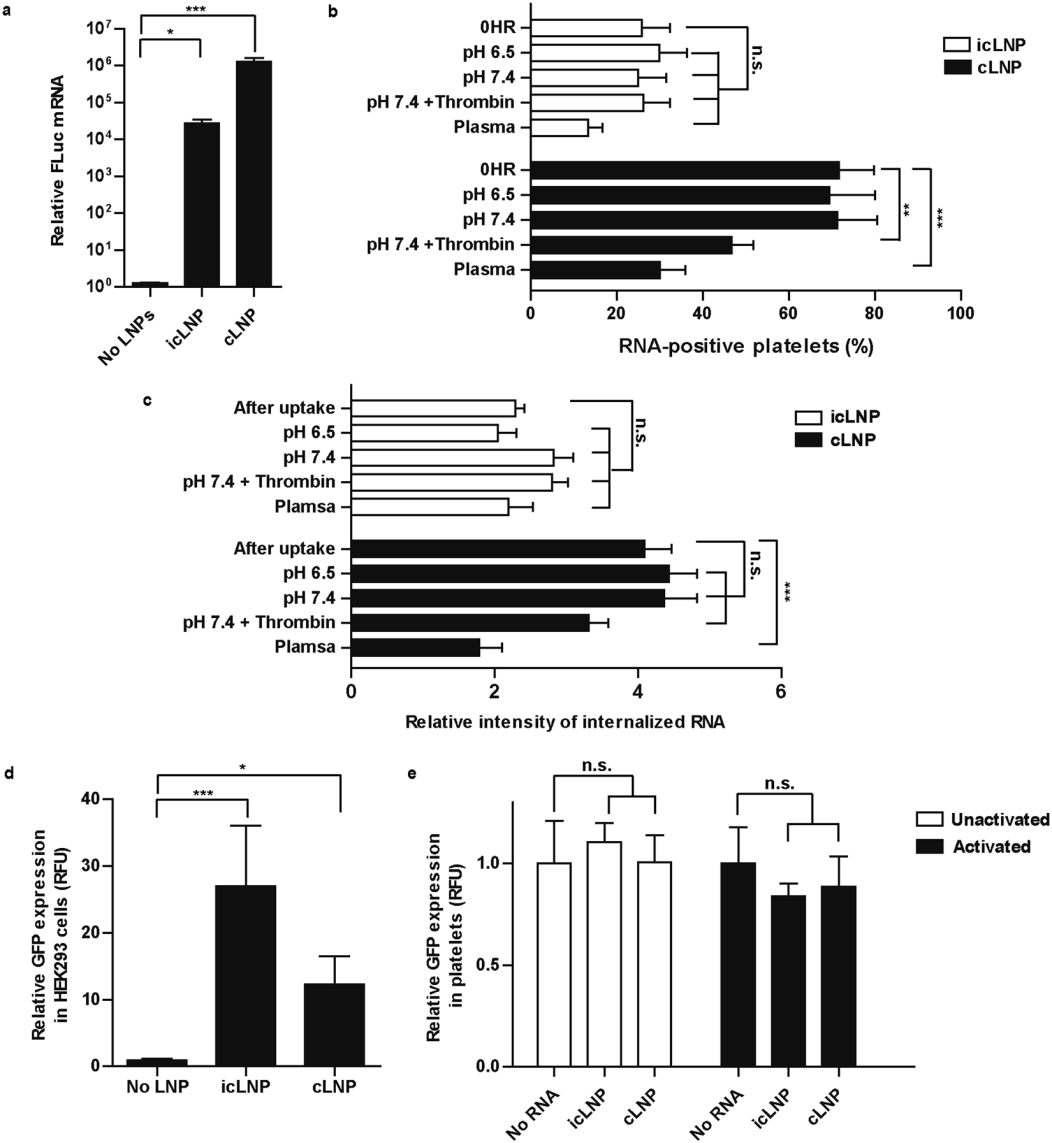
icLNPs and cLNPs are stable in resting platelets but the delivered mRNA is not translated. (**a**) Quantifying the amount of mRNA using qPCR after excess LNPs were removed (n = 12). (**b**) Quantifying the number of Cy5-positive platelets 4 h after excess LNPs were removed and platelets were resuspended in Tyrode’s buffer at pH 6.5 or pH 7.4 or in plasma, using flow cytometry (n = 5). (**c**) Assessing the amount of RNA that remains inside platelets during storage for 2 h under different conditions by quantifying biotin-labelled RNA in confocal immunofluorescence images of platelets (n = 5). (**d**) HEK293 cells (n = 8) and (**e**) platelets (n = 5) were transfected with LNPs containing GFP mRNA and after 24 h protein expression was quantified by measuring green fluorescence. N.S. means not significant. Error bars represent SEMs. *P < 0.05, **P < 0.01, ***P < 0.001.

As the flow cytometry and confocal microscopy experiments could not distinguish between single nucleotides and intact mRNA, the amount of mRNA was measured by quantitative PCR (qPCR). Only icLNPs and cLNPs were assessed, because they were the only formulations internalized by platelets. A short fragment of mRNA, 127 base pairs long, could be detected in platelets with icLNPs (P = 0.0429) and cLNPs (P < 0.0001) (Fig. 6a). The stability of the mRNA delivered by these formulations was next examined by flow cytometry (Fig. 6b). Four hours after removing excess LNPs, the percentage of Cy5-positive platelets with icLNPs was unaltered in resting or activated platelets stored in buffer or plasma. The percentage of Cy5-positive platelets with cLNPs decreased when platelets were reconstituted in plasma (P < 0.0001), and when activated with thrombin at pH 7.4 (P = 0.0044), but was unchanged when platelets were stored at pH 7.4 in the absence of any agonist. Confocal microscopy was then used to determine whether the fluorescence remained internalized within the platelets under these conditions (Fig. 6c, Supplementary Fig. 3). After removing excess icLNPs, there was no significant difference in the fluorescence intensity inside of the platelets in any storage medium, compared to immediately after uptake. After removing excess cLNPs, the internal fluorescence intensity decreased with storage in plasma (P = 0.0005), but not in buffer at pH 6.5 or 7.4.

The ability of platelets to translate the delivered mRNA was then tested. To ensure the LNPs were functional, human embryonic kidney cells 293 (HEK293) were first transfected with icLNPs and cLNPs containing green fluorescent protein (GFP) mRNA. While HEK293 cells expressed GFP (P = 0.0228 for cLNP, P = 0.0003 for icLNP) (Fig. 6d), platelets with icLNPs or cLNPs did not (Fig. 6e).

**Figure 7:**
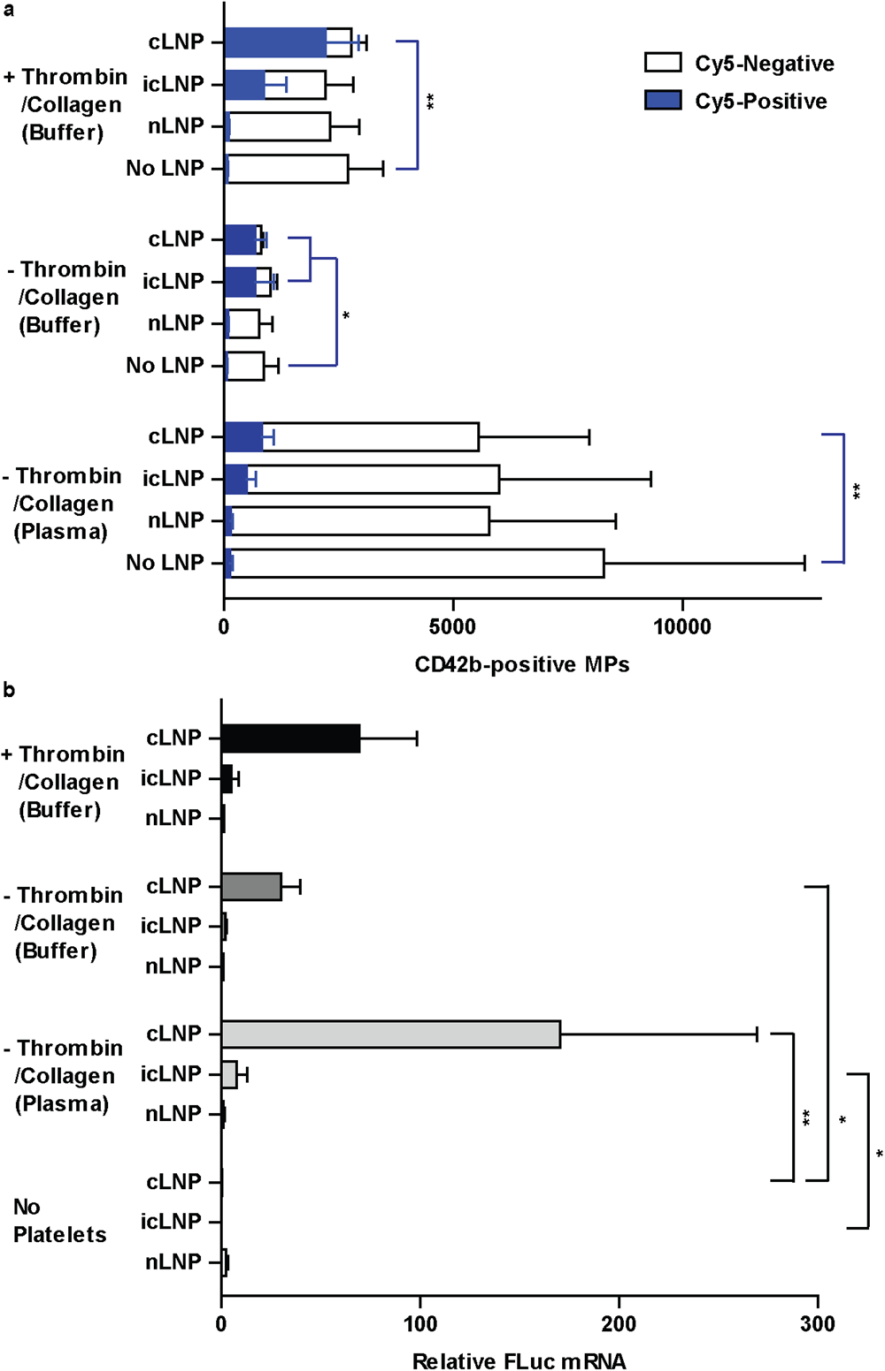
mRNA delivered by LNPs is released in platelet MPs. MPs were isolated from platelets prepared with LNPs and resuspended in either Tyrode’s buffer, with or without activation, or in plasma without activation. (**a**) The number of Cy5-positive and Cy5-negative MPs from 1 × 10^8^ platelets, quantified using flow cytometry (n = 6). (**b**) The amount of FLuc mRNA in all MPs from 1 × 10^8^ platelets, quantified by qPCR (n = 7). Error bars represent SEMs. *P < 0.05, **P < 0.01.

To test if platelets released the delivered mRNA in MPs, flow cytometry was used to detect whether Cy5 localized to MPs ranging from 100 nm to 400 nm in diameter (Supplementary Fig. 4) and expressing CD42b, a marker of platelet MPs. MPs were formed by platelets activated by the oxidative and mechanical stress induced by *ex vivo* isolation and storage of the platelets^20,21^, or by treatment with thrombin and collagen. When platelets were not treated with specific agonists, a significant increase in Cy5-mRNA was detected in MPs from platelets prepared with cLNPs and stored in either buffer (P = 0.0219) or plasma (P = 0.0091) (Fig. 7a). A significant increase in mRNA could also be detected under these conditions using qPCR (P = 0.0225 in buffer, P = 0.0056 in plasma) (Fig. 7b). In MPs from platelets treated with thrombin and collagen, a significant increase in Cy5-positive MPs was detected in platelets prepared with cLNPs (P = 0.0105), and more than 50% of platelet MPs were Cy5-positive (Fig. 7a). An increase could not be conclusively demonstrated by qPCR. When platelets were prepared with icLNPs and not intentionally activated, there a significant increase in Cy5-postiive MPs from platelets stored in buffer (P = 0.0437) (Fig. 7a). The qPCR analysis did not demonstrate a conclusive increase. When platelets were prepared with icLNPs and stored in plasma, a significant increase in mRNA from MPs was detected by qPCR (P = 0.0114) (Fig. 7b), but under these conditions there was no conclusive increase in Cy5-positive MPs.

## Discussion

Here, we describe a method for the direct delivery of mRNA to platelets. Platelets efficiently internalized mRNA encapsulated within nanoparticles containing either a cationic lipid or an ionizable cationic lipid, by a dynamin-dependent mechanism of endocytosis. Platelets retained their activity after they were prepared with LNPs and the mRNA delivered was released in platelet MPs under specific conditions.

Platelets internalized mRNA only when prepared with icLNPs and cLNPs, but not nLNPs or Lf. In our experiments, we found that icLNPs and cLNPs were smaller relative to the Lf-mRNA complexes, which may have facilitated increased uptake by platelets^22^. It is possible larger lipid-based materials require longer than 2 hours for endocytosis^14^. In other mammalian cells, uptake of polymeric nanoparticles 100 to 150 nm in diameter is generally faster than uptake of 500 nm particles, unless uptake occurs by phagocytosis^23,24^. The inability of amiloride to prevent uptake of LNPs in platelets, including Lf, suggests uptake is not occurring by phagocytosis. To more conclusively determine the role of LNP size on uptake by platelets, direct comparisons between LNPs from the same class but with varying diameters would be required.

While nLNPs were similar in size compared to icLNPs and cLNPs, there was poor uptake of mRNA encapsulated in nLNPs. This was expected, as a cationic lipid is necessary for efficient encapsulation of mRNA^25,26^. Raising the pH during uptake from 6.5 to 7.4 reduced the percentage of Cy5-positive platelets and the fluorescence intensity within icLNPs, but not cLNPs, likely because icLNPs contain an ionizable lipid with a pKa of 6.7, while cLNPs have a pH-insensitive cationic lipid. Together, the results suggest that a cationic lipid is important for uptake in platelets, as it is in other mammalian cells^11^.

When platelets were incubated with icLNPs or cLNPs in plasma, the percentage of Cy5-positive platelets and the fluorescence intensity for both formulations was reduced. Plasma proteins can bind to LNPs, which affects their ability to be internalized by cells^22^. While the effect of incubating platelets in buffer before reconstituting in plasma was not fully characterized in this paper, we have previously shown that platelets prepared with protein-loaded LNPs can still clot blood *in vitro* after short periods of storage in buffer^27^. Here, the icLNPs did not activate the platelets or affect their ability to aggregate or spread. Only the cLNPs induced alpha-granule release, indicated by P-selectin expression, and caused faster thrombin generation, without impairing aggregation or spreading. Future *in vivo* studies will be required to determine whether platelets prepared with mRNA-LNPs maintain their ability to contribute to hemostasis. However, as platelets were only activated by cLNPs, these effects may be reduced by optimizing the LNP formulation, perhaps by using additional high-throughput screening, as has been done to optimize nucleic acid formulations for other mammalian cells^28,29^.

Platelet MPs contain endogenous miRNA and mRNA^3,30^. Here we show that Cy5 from mRNA delivered by cLNPs was released in the MPs of platelets activated by thrombin and collagen, while mRNA delivered using either cLNPs or icLNPs was found in MPs produced in the absence of a specific platelet agonist. It has previously been demonstrated that platelet MPs can be internalized and alter protein expression in recipient cells^3,31^, highlighting the potential for developing cell therapy approaches that use LNP-treated platelets as delivery vehicles for exogenous mRNA.

Since translation of the delivered mRNA was not observed, additional characterization of the RNA following uptake into the platelets is needed. It is unclear whether the absence of translation was due to degradation of the mRNA, differences in how translation is regulated in platelets compared to other mammalian cells, or whether the mRNA was not delivered to the appropriate location in the platelet. Although we amplified a fragment of mRNA that was 127 base pairs long, we were not able to determine how much intact, functional mRNA was present in platelets and MPs. While platelets express proteins during storage and activation, the factors that regulate translation in platelets are less characterized compared to other mammalian cells^32–34^. Additionally, efficient protein expression or knockdown of target protein with siRNA delivery for other transfected cells is often limited by the release of transfected RNA from endosomes and disassembly of the nucleic acid-cationic complex^11,13,35^. In our studies, platelets were only stained with a membrane marker. Analyzing co-localization with markers of organelles by super-resolution methods or transmission electron microscopy could help determine the subcellular localization of the delivered mRNA^36^. To achieve protein expression in platelets, it may be necessary to optimize both the mRNA and the LNP transfection agent in the future.

In summary, this paper demonstrates a method for efficient delivery of mRNA to platelets, with minimal platelet activation and impact on platelet function *in vitro*. This is a first step towards the direct genetic modification of transfusable platelets, to create transfusable platelets that can perform new or enhanced functions *in vivo*. This work highlights the need for developing LNP formulations and transcripts specific to platelets, likely using high-throughput screening^28,29^ that starts with the icLNPs and cLNPs tested here, in order to identify reagents that enable *de novo* protein synthesis in platelets.

## Materials and Methods

### Preparing four classes of lipid nanoparticles

LNPs lacking cationic lipids (nLNPs) were prepared using a protocol previously described^16^. A thin film of 12 µmol of lipids, consisting of egg-phosphatidylcholine:cholesterol:1,2-distearoyl-sn-glycero-3-phosphoethanolamine-N-[amino(polyethylene glycol)-2000] (ePC:chol:DSPE-PEG2000; 58.9:39.6:1.5 molar ratio;) was rehydrated with deionized water, extruded ten times through a 200 nm filter using a LIPEX extruder (Northern Lipids Inc.), and lyophilized. The lyophilized lipid was rehydrated with deionized water to 16 mM and extruded through a 200 nm filter using a Mini-extruder (Avanti Polar Lipids) eleven times. The formed liposomes were mixed with mRNA (25 µg mL^−1^) in tris-buffered saline (TBS; 50 mM Tris-HCl, 150 mM NaCl) to a final concentration of 12 mM lipids, and the mixture was freeze-thawed once to encapsulate the mRNA.

LNPs containing ionizable cationic lipids or cationic lipids (icLNPS and cLNPs, respectively) were prepared using a method previously described for formulating siRNA-encapsulated LNPs^18^. Briefly, 3 µmol of lipids, consisting of cholesterol, 1,2-dioleoyl-sn-glycero-3-phosphocholine, N-[(methoxy poly(ethylene glycol)2000)carbamoyl]-1,2-dimyristyloxlpropyl-3-amine (PEG-c-DMA) and either 2,2-dilinoleyl-4-(2-dimethylaminoethyl)-[1,3]-dioxolane (DLin-KC2-DMA) for icLNPs or 1,2-dioleoyl-3-trimethylammonium-propane (DOTAP) for cLNPs were mixed in a 10:38.5:1.5:50 molar ratio in an ethanol-sodium citrate solution (30% v/v, 50 mM sodium citrate, pH 4) to a final concentration of 12 mM before extruding three times through a 200 nm filter using a LIPEX extruder. The lipids were mixed with mRNA (25 µg mL^−1^) to a final concentration of 6 mM lipids and equilibrated for 30 min at 22 °C. The LNPs were then dialyzed against phosphate-buffered saline (PBS) for 24 h.

Lipofectamine-mRNA complexes were formed by diluting Lipofectamine MessengerMax (5% v/v) and mRNA (25 µg mL^−1^) in PBS and incubating for 10 min at 22 °C.

Nanoparticle size was measured using dynamic light scattering with Zetasizer Nano ZPS (Malvern).

### Preparing washed platelets

Approval for the study was obtained from the ethics boards of the University of British Columbia (UBC) and the Canadian Blood Services (CBS), in accordance with the UBC and CBS Research Ethics Boards’ guidelines. Informed, signed consent was obtained from healthy volunteers before their blood was collected. Platelet rich plasma (PRP) was obtained from pooled donor plasma generated by the CBS netCAD facility or isolated from citrated (0.105 M) whole blood by centrifuging at 100 × *g* for 20 min and collecting the top 75% (v/v) of the upper layer. The source of PRP was kept consistent for a given experiment. PRP was then centrifuged at 250 × *g* for 20 min and the platelet poor plasma was removed. The platelets were washed once in a citrate-glucose-saline buffer (CGS; 120 mM NaCl, 30 mM D-glucose, 11 mM trisodium citrate, pH 6.5) and once in Tyrode’s-HEPES buffer (1.8 mM CaCl_2_, 1.1 mM MgCl_2_, 2.7 mM KCl, 137 mM NaCl, 0.4 mM NaH_2_PO_4_, 10 mM HEPES, 5.6 mM D-glucose, pH 6.5) at 250 × *g* for 10 min. Prostaglandin E1 (PGE1; 10 µM) was added at all wash steps. Washed platelets were suspended in Tyrode’s-HEPES at a concentration of 200 × 10^9^ platelets L^−1^.

### Generating biotinylated mRNA

A DNA template containing a T7 promoter, an EMCV internal ribosome entry site, the protein coding sequence for luciferase, and a polyadenylate tail was first generated by cloning the Firefly Luciferase (FLuc) coding sequence from pEJ3 (a gift from E. Jan, UBC) into the MCS of pT7CFE1-CHis using Pst1 and PvuII. PCR using primers forward 5′-GGC CTC TTC GCT ATT ACG C-3′ and reverse 5′-CGA GGA AGC CCG GAT ATA GT-3′ were used to amplify the sequence for FLuc. Biotinylated mRNA was generated by incubating 50 mM PCR template with ATP, CTP and GTP (1 mM each), UTP (6.5 mM), Biotin-16-UTP (3.5 mM), and 10% (v/v) each of T7 RNA polymerase and transcription buffer (MEGAscript T7 Transcription Kit, ThermoFisher) for 3 h at 37 °C, then 0.1 U µL^−1^ TURBO DNase I for 1 h. RNA was purified by precipitating in 2.5 M lithium chloride overnight at 4 °C, centrifuging for 15 min at 21,000 × *g*, washing the pellet with 70% (v/v) ethanol for 5 min at 21,000 × *g*, and resuspending in water at 1 mg mL^−1^.

### Treating platelets with LNPs

LNPs (1:100 v/v) or free RNA (250 ng mL^−1^) were added to washed platelets, and the mixture was incubated for 2 h at 22 °C. For flow cytometry and translation experiments, LNPs contained capped, cyanine 5-UTP and 5-methoxy-UTP-labelled (Cy5) mRNA encoding either FLuc GFP. For all other experiments, LNPs contained *in vitro* transcribed biotin-UTP-labelled FLuc mRNA. Unless otherwise stated, uptake was performed in Tyrode’s-HEPES buffer (pH 6.5). To remove excess LNPs, platelets were centrifuged for 15 min at 250 × *g*, washed once with CGS, once with Tyrode’s-HEPES, and resuspended in Tyrode’s-HEPES, modified Tyrode’s-HEPES (134 mM NaCl, 3 mM KCl, 0.3 mM NaH_2_PO_4_, 2 mM MgCl_2_, 5 mM HEPES, 5 mM D-glucose, 12 mM NaHCO_3_, 3.5 mg mL^−1^ BSA, pH 7.4) or citrated human plasma (Affinity Biologicals) to 200 × 10^9^ platelets L^−1^.

### Quantifying uptake of LNPs and alpha granule release by flow cytometry

To inhibit uptake of LNPs, platelets were pre-incubated for 30 min with dynasore (50 µM), sodium azide (50 µM), cytochalasin D (10 µM), or amiloride (1 mM) before adding LNPs. Platelets were stained with anti-human CD42b-FITC antibody (1 µg mL^−1^; ThermoFisher) for 30 min and analyzed by flow cytometry using a FACSCalibur (BD Biosciences) and CellQuest Pro software. Platelets were identified using forward and side scatter, as well as CD42b-FITC staining. To quantity alpha granule release by flow cytometry, platelets were incubated with LNPs for 1.5 h and stained with anti-human CD42b-FITC and CD62-allophycocyanin (1 µg mL^−1^, ThermoFisher) for 30 min.

### Quantifying uptake of LNPs by confocal microscopy

Platelets were directly fixed with 4% paraformaldehyde (w/v) and adhered onto poly-L-lysine-coated cover slips. Cover slips were washed once with PBS, treated with 0.5% Triton X-100 for 20 min, and then blocked with 10% (v/v) goat serum in PBS overnight at 4 °C. Platelets were stained with mouse anti-human CD42b antibody (2 µg mL^−1^, ThermoFisher) for 1.5 h at room temperature, washed three times with PBS, and then stained with streptavidin-FITC (1 µg mL^−1^, ThermoFisher) and Brilliant Violet 421 goat anti-mouse secondary antibody (100 ng mL^−1^, BioLegend) for 1 h. After three PBS washes, slides were mounted and images acquired using an Olympus FV1000 confocal microscope and Olympus FluoView software. The relative amount of mRNA was quantified by measuring the pixel intensity within the ring of CD42b staining for approximately 20 platelets using ImageJ software, normalizing to untreated platelets.

### Measuring thrombin generation

Following removal of excess LNPs, platelets were resuspended in citrated human plasma containing a fluorescent thrombin substrate (1 µM, Boc-Asp(OBzl)-Pro-Arg-MCA). Following addition of calcium buffer (40 mM CaCl_2_, 90 mM NaCl), platelets were incubated at 37 °C and fluorescence was monitored using a plate reader (Tecan Genios). The lag time was determined as the time at which thrombin generation began.

### Measuring platelet spreading

Platelets were placed on collagen-coated coverslips and fixed immediately with 4% paraformaldehyde or incubated with thrombin (2 U mL^−1^) at 37 °C for 1 h before fixing. After fixing, platelets were permeabilized and blocked overnight as described above. Platelets were stained for 1.5 h with an F-actin probe (1:50,000 v/v, ActinGreen 488 ReadyProbes, ThermoFisher) before washing and mounting. To quantify platelet area, ImageJ was used to determine the surface area of individual platelets, with one to two dozen platelets quantified per condition.

### Measuring platelet aggregation

Washed platelets were obtained by running PRP over a Sepharose 2B column equilibrated in modified Tyrode’s buffer. Platelet aggregation was measured using a lumiaggregonometer (Chrono-log) after the addition of ADP (10 µM), human fibrinogen (250 µg mL^−1^), and LNPs or free RNA either immediately after collection of washed platelets or 2 h after treatment with LNPs.

### Quantifying protein expression

Platelets were suspended in M199 media at a concentration of 200 × 10^10^ platelets L^−1^ and activated with thrombin (52 U mL^−1^) and collagen (10 µg mL^−1^) or left untreated. After 16 h at 37 °C, platelets were centrifuged for 20 min at 250 × *g*, washed once in PBS, and resuspended in PBS. HEK293 cells (ATCC) were grown in FluoroBrite Dulbecco’s Modified Eagle Medium, supplemented with Penicillin/Streptavidin (5% v/) and heat-inactivated fetal bovine serum (5% v/v), and transfected with icLNPs or cLNPs (0.5 µg mRNA mL^−1^). After 24 h, cells were washed once with PBS, treated with 12.5 µg mL^−1^ trypsin-EDTA for 5 min and diluted with an equivalent volume of PBS. To measure protein expression, the fluorescent intensity (λEx/ λEm = 485/535 nm) was measured using a Tecan Genios plate reader.

### Isolating platelet MPs

After removing excess LNPs, platelets were suspended in modified Tyrode’s buffer (pH 7.4) or plasma. Only platelets in buffer were intentionally activated by incubating with calcium chloride (5 mM), thrombin (2 U mL^−1^) and collagen (10 µg mL^−1^) for 2 h at 37 °C. Platelets were then centrifuged for 20 min at 250 × *g* and the resulting supernatant centrifuged for 10 min at 3,200 × *g* to clear platelet debris. The supernatant was removed and centrifuged for 90 min at 21,000 × *g* to isolate MPs. The MP pellet was resuspended in PBS before flash-freezing in liquid nitrogen. After thawing, MPs were stained with anti-human CD42b-FITC for 30 min at 4 °C before analysis by flow cytometry using a CytoFLEX Flow Cytometer (Beckman Coulter) and CytExpert software. Gating was performed using CD42b staining.

### Quantifying mRNA by qPCR

Platelets or MPs were resuspended in Trizol reagent. RNA was extracted using the manufacturer’s protocol, digested with a TURBO DNA-free Kit (ThermoFisher), reverse transcribed into cDNA using oligo-D(T) primers and M-MLV reverse transcriptase, and detected using qPCR with gene-specific primers and SYBR green reagents. RNA was quantified using 2^−ΔΔCt^, with GAPDH as an internal control for platelets and exogenous GFP mRNA added as an internal control for MPs.

### Statistical analysis

All experiments were repeated a minimum of 4 times, with sample sizes indicated in each figure. All values are expressed as mean ± SEMs. A Shapiro-Wilkes test was used to test for normality. When data was normally distributed, comparisons were made using a paired and two-tailed t-test if only 2 groups were compared and by a one-way ANOVA, with a Bonferroni post-hoc test, when more than 2 groups were compared. If data was not normally distributed, comparisons were made by a Friedman test with a Dunn’s multiple comparisons post-hoc test. A *P*-value < 0.05 was considered significant.

## Supplementary information

Supplementary Information
