# Supplementary material for: Delivery of mRNA to platelets using lipid nanoparticles
Source: Sci Rep. 2019 Jan 24;9:552. doi: 10.1038/s41598-018-36910-2 (PMC6345896; doi:10.1038/s41598-018-36910-2)
Supplement: Supplementary file 1 — Supplementary Information [file 41598_2018_36910_MOESM1_ESM.pdf]

Delivery of mRNA to platelets using lipid nanoparticles

S. Novakowski[1,2], K. Jiang[1], G. Prakash[1], C. Kastrup[1,2*]

[1]*Michael Smith Laboratories, University of British Columbia, Vancouver, BC, Canada.*
[2]*Department of Biochemistry and Molecular Biology, University of British Columbia, Vancouver, BC, Canada.*

[*]Corresponding author:
Christian Kastrup, PhD
Michael Smith Laboratories
University of British Columbia
275 - 2185 East Mall
Vancouver, BC Canada V6T 1Z4
Phone: 604 827 3749
Email: ckastrup@msl.ubc.ca

Supplementary information:

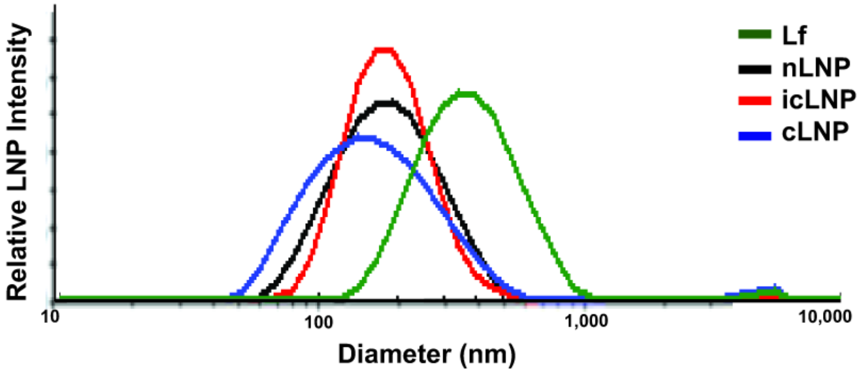

Fig S1. LNPs have similar size characteristics except for Lf. Dynamic light scattering was used to measure the average diameter of the different formulations. Representative plots of 3 different experiments are shown.

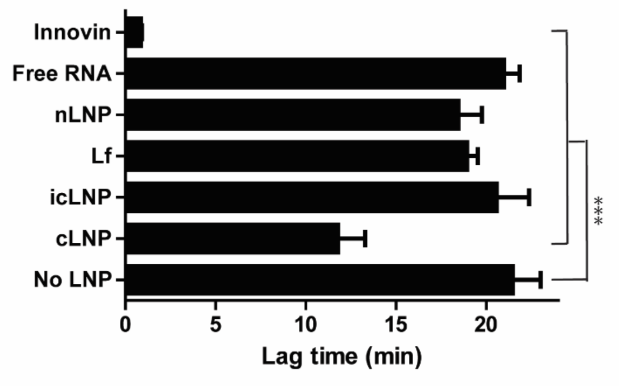

Fig S2. cLNPs induce thrombin generation in the absence of platelets. The time to generate thrombin following treatment with LNPs was quantified using a fluorescent thrombin substrate (n=4). ***$P <$ 0.001

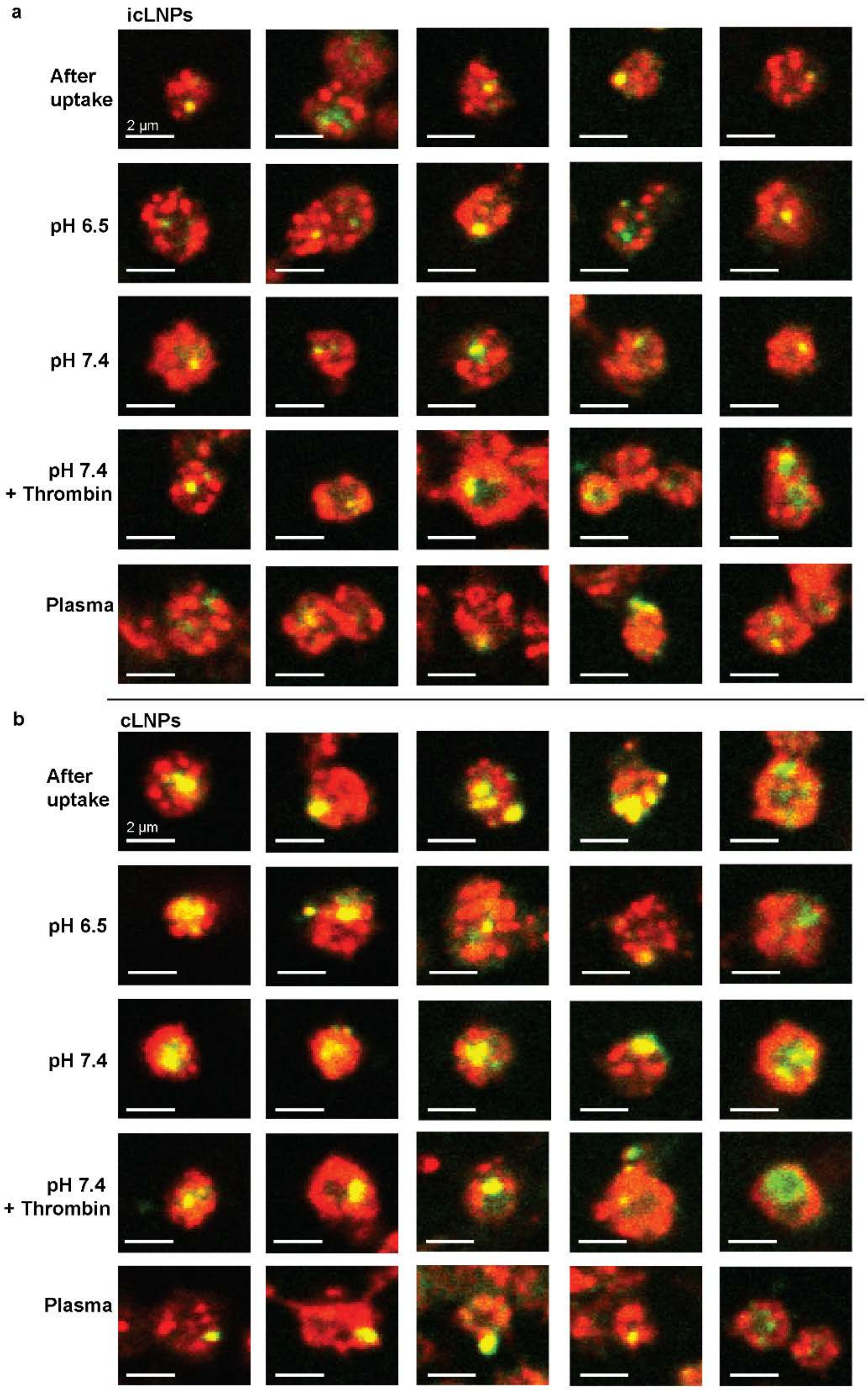

Fig S3. Both icLNPs and cLNPs remain internalized after uptake unless platelets are stored in plasma. Confocal immunofluorescence microscopy of platelets (red) transfected with a) icLNPs and b) cLNPs containing biotin-labelled RNA (green) 2 h after removal of LNPs. Representative images for 5 different donors are shown. All scale bars indicate 2 µm.

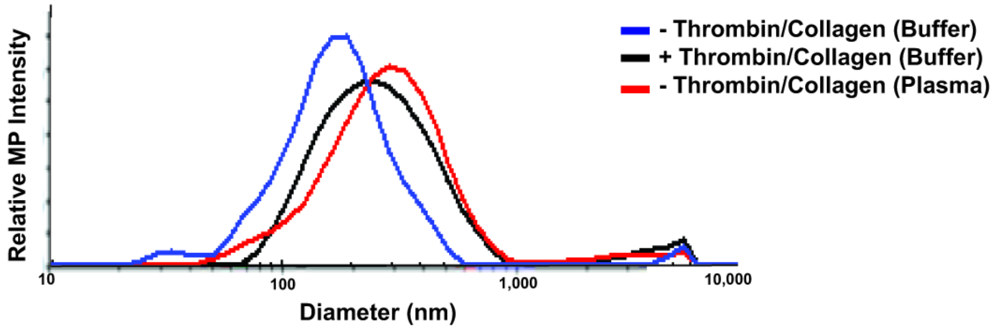

Fig S4. Platelet microparticles range from 100 nm to 400 nm. Dynamic light scattering was used to measure the size of microparticles released from platelets. Representative plots of 3 different donors are shown.
